# Field-Grown Rice Plants Become More Productive When Exposed to Artificially Damaged Weed Volatiles at the Seedling Stage

**DOI:** 10.3389/fpls.2021.692924

**Published:** 2021-07-12

**Authors:** Kaori Shiojiri, Rika Ozawa, Masayoshi Uefune, Junji Takabayashi

**Affiliations:** ^1^Department of Agriculture, Ryukoku University, Otsu, Japan; ^2^Center for Ecological Research, Kyoto University, Otsu, Japan; ^3^Department Agrobiological Resources, Faculty of Agriculture, Meijo University, Nagoya, Japan

**Keywords:** rice, artificially damaged plant volatiles, weeding, green leaf volatiles, terpenoids, *Oryza sativa* subsp. *japonica*

## Abstract

It is known that undamaged plants that have been exposed to volatiles from damaged con- or heterospecific plants become more resistant against herbivores. This is one of the plants’ induced resistant responses against herbivores. To test whether this response can be used for rice production, we conducted the following experiments over 2 years (2012 and 2013). Rice seedlings were first planted in the rice seedling bed for 2 weeks in early May. There, half of the rice seedlings were exposed to artificially damaged weed volatiles three times for 12 days (treated plants). Weeds were randomly collected from the areas that were >100 m away from the seedling bed and the rice paddy fields. The remaining seedlings were not exposed (control plants). In the middle of May, bunches (ca. three seedlings per bunch) were transplanted to the rice paddy field. In July, leaf damage was observed. The total number of leaves in the treated and control plants was not significantly different. In contrast, the total number of damaged leaves in the treated plants was significantly lower than that in the control plants. In September, rice grains were harvested. The average weight of a rice grain from the treated and control plants was not significantly different. However, the weight of grains per bunch of treated plants was significantly higher than that of control plants; this indicated a significant increase of the number of grains by 23% in 2012 and by 18% in 2013 in the treated plants compared to that in the control plants. The volatiles emitted from the weeds included monoterpenoids (40.4% in total), green leaf volatiles (46.5%), short-chain alcohols (5.3%), short-chain ketone (5.4%), short-chain acetate (0.5%), short-chain aldehyde (1.1%), and hydrocarbon (0.7%). These results suggest that exposure of volatiles from artificially damaged weeds to rice seedlings has the potential to increase rice production.

## Introduction

In response to damages caused by herbivorous arthropods, plants start emitting herbivory-induced plant volatiles (HIPVs) (Takabayashi and Shiojiri, 2019). When uninfested conspecific plants received HIPVs, they become more defensive against herbivores [for review see Karban et al. (2014) and Yoneya and Takabayashi (2016)]. Besides responding to HIPVs, plants also respond to volatiles from artificially damaged plants. These phenomena are called “priming of plant resistant by plant volatiles.” Under laboratory conditions, green leaf volatiles (GLVs), which are one of the common volatiles emitted from herbivore-damaged and artificially damaged plants, have been shown to induce resistant responses in *Arabidopsis thaliana*, *Citrus jambhiri* (rough lemon), *Zea mays* (corn), and *Nicotiana attenuata* (wild tobacco) (Bate and Rothstein, 1998; Gomi et al., 2003; Engelberth et al., 2004; Farag et al., 2005; Kishimoto et al., 2005, 2006; Paschold et al., 2006; Yamauchi et al., 2018). When undamaged pyrethrum plants (*Chrysanthemum cinerariifolium*) were exposed to a blend of volatiles (GLVs and a sesquiterpene) emitted from artificially damaged conspecific leaves, the amount of pyrethrin in the exposed plants increased significantly compared to that in plants that were not exposed (Kikuta et al., 2011). Under field conditions, sagebrush plants (*Artemisia tridentata*) exposed to volatiles from artificially damaged conspecific plants suffered less damage compared to unexposed conspecifics (Karban et al., 2006). The priming is also observed between heterospecific plants (Farmer and Ryan, 1990; Oudejans and Bruin, 1995; Karban et al., 2003). Under laboratory conditions, exposure of undamaged cucumber plants (*Cucumis sativus*) to HIPVs from spider mite-infested Lima bean (*Phaseolus lunatus*) plants resulted in the attraction of predatory mites that preyed on spider mites (Oudejans and Bruin, 1995). Under field conditions, Karban et al. (2003) showed an induced resistance in tobacco when sagebrush neighbors were clipped either with scissors or damaged with herbivores.

Weeding is a common practice in agricultural fields. During this practice, a large amount of artificially damaged weed volatiles is spread over the surrounding areas, including agricultural fields, thus exposing the surrounding crops to artificially damaged weed volatiles. After weeding, the exposed crops are expected to be more resistant than non-exposed crops to herbivores (priming of plant resistance by volatiles from heterospecific plants). Recently, this possibility was demonstrated in field-grown black soybeans and yellow soybeans (*Glycine max*); when young soybean plants were exposed to artificially damaged goldenrod volatiles, both plants and their grains became more resistant against herbivores (Shiojiri et al., 2017, 2020).

Sticky rice (*Oryza sativa* subsp. *japonica*) is an important food source worldwide. In Japan, weeding around rice paddy fields is conducted in spring to remove Gramineae weeds, which host many insect pests of rice plants. This weeding results in the rice seedlings being exposed to weeding-related volatiles. In the present study, we investigated whether such exposure affected rice grain production in pesticide-free rice paddy fields. We discussed the potential of using weeding-related volatiles in yield control in rice production.

## Materials and Methods

### Experimental Conditions

Field experiments were carried out during 2012 and 2013 in pesticide-free commercial rice paddy fields owned by a farmer in Makino, Shiga, Japan. Rice plants (*O. sativa* subsp. *japonica* var. Koshihikari) were cultivated in eight paddy fields. Approximately 15,000 rice bunches (2–3 plants per bunch) were planted in each paddy field (20 m × 50 m). Four paddy fields (A, B, C, and D) were set together, while the other four (E, F, G, and H) were set at another site; the two sites were located ca. 200 m away from each other. At each site, we established treatment and control fields. A seedling bed (1 m × 15 m) was used to grow rice seedlings before transplanting them to the paddy fields.

### Odor Sources

Several species of weeds growing in areas that were >100 m away from the rice paddy fields and from the rice seedling bed were used as odor sources. In both years, we observed weeds belonging to more than six families (including Asteraceae, Equisetaceae, Caryophyllaceae, Fabaceae, Poaceae, and others) and identified the following four major species: *Picris hieracioides* var. *glabrescens* (Asteraceae; Cichorieae), *Artemisia indica* Willd. var. *maximowiczii* (Asteraceae), *Equisetum arvense* (Equisetaceae), and *Trifolium repens* (Fabaceae; Trifolieae). We randomly collected weeds (5 kg) in the areas and divided them equally into ten portions. Each portion was placed in a mesh bag (45 cm × 35 cm). The composition of weed species used for the experiments was the same during the 2-year experiments. The used weeds were perennial plants and no herbicides were used in two consecutive years.

### Experimental Procedures

To facilitate the exposure experiments, we conducted experiments in a rice seedling bed, because plant defenses for future growth are often developed at the seedling stage (Barton and Koricheva, 2010). We divided the seedling bed into two parts (the control area and the exposed area) with a polycarbonate board (ca. 1 m high and 2 m long) to prevent weed volatiles from reaching the untreated seedlings. When the seedlings in the bed were ca. 3–5 cm high, half of them (ca. 150,000 seedlings) were exposed to weeding-related volatiles. Ten mesh bags containing cut weeds (ca. 500 g per bag) were hung equidistantly in the exposed area of the seedling bed. The cut weeds were replaced with new ones every 4 days for 12 days. In 2012, after the exposure, the treated and control rice seedlings (ca. 10 cm high) were transplanted into rice paddy fields: the exposed seedlings were planted in A and H fields, and the control seedlings were placed in D and E fields. B, C, F, and G fields were used for other experiments. In 2013, the exposed seedlings were placed in B, D, E, and G fields, and the control seedlings were placed in A, C, F, and H fields.

Approximately 50 days after the transplantation, we collected the leaves from 15 treated and 15 control rice bunches randomly selected [except for field B (treated) in 2013, *N* = 14] to evaluate the number of damaged leaves on each paddy field.

In the rice paddy field used in this study, we observed commonly found herbivorous arthropods, such as rice leaf beetles (*Oulema oryzae*), rice leaf folders (*Cnaphalocrocis medinalis*), rice green caterpillars (*Naranga aenescens*), and Japanese grasshoppers (*Oxya yezoensis*). Rice leaf beetles and rice leaf folders made similar fed-edge leaving leaf veins after herbivory. Rice green caterpillars and Japanese grasshoppers made different fed-edge because they did not leave leaf veins. In the field observation, we could not identify the herbivore species based on the fed-edges on damaged leaves. The two types of fed-edges were evaluated together as damaged leaves.

After harvest, we measured the weight of rice grains from 15 treated, and 15 control rice bunches randomly selected. We weighted 100 grains per bunch. Because each bunch had approximately 8,000–10,000 grains, we were unable to count the number of grains per bunches on site. The detailed procedure of the experiments is presented in Supplementary Table 1.

### Chemical Analysis

On May 12, 2013, in order to analyze the chemical structure of weeding-related volatiles, we used ca. 100 g of cut weeds per mesh bag from five randomly selected mesh bags that were used for the exposure experiments. After volatile collection, we returned them to the mesh bag. We placed the samples in polyethylene terephthalate (PET) bags (180 mm × 250 mm) (Mitsubishi Gas Chemical Company, Inc., Tokyo, Japan). To collect volatiles, we sent air (100 mL/min airflow) into each PET bag containing weeds. At the outlet of each bag, we set a tube containing Tenax-TA (3.0 mm internal diameter, 160 mm long with 100 mg, GL Sciences Inc., Tokyo, Japan) to collect volatiles for 1 h. We analyzed the collected volatiles by gas chromatography/mass spectrometry (GC/MS) (GC: Agilent 6890 with an HP-5MS capillary column: 30 m long, 0.25-mm i.d., and 0.25-μm film thickness; MS: Agilent 5973 mass selective detector, 70 eV with helium as the carrier gas; Agilent, Santa Clara, CA, United States) equipped with a thermal desorption cold trap injector (TCT; CP4010; Chrompack, Middelburg, Netherlands). We programmed the oven temperature of the gas chromatograph to increase from 40°C (5 min hold) to 280°C at 15°C/min. We identified the compounds by comparing their mass spectra with those of compounds from the Wiley7N database (John Wiley & Sons Inc., Hoboken, NJ, United States) and their retention times and mass spectra with those of synthetic compounds.

### Statistical Analyses

The effects of treatment, year, and their interaction on the number of leaves and grain weight per bunch were analyzed with the least square mixed model after Box-Cox transformation in JMP version 14.2.0 (SAS Institute, 2018). The rice paddy field was a random effect in all models. The weight of 100 grains was analyzed with a *t*-test in JMP version 14.2.0.

## Results

### Leaf Damage

**FIGURE 1:**
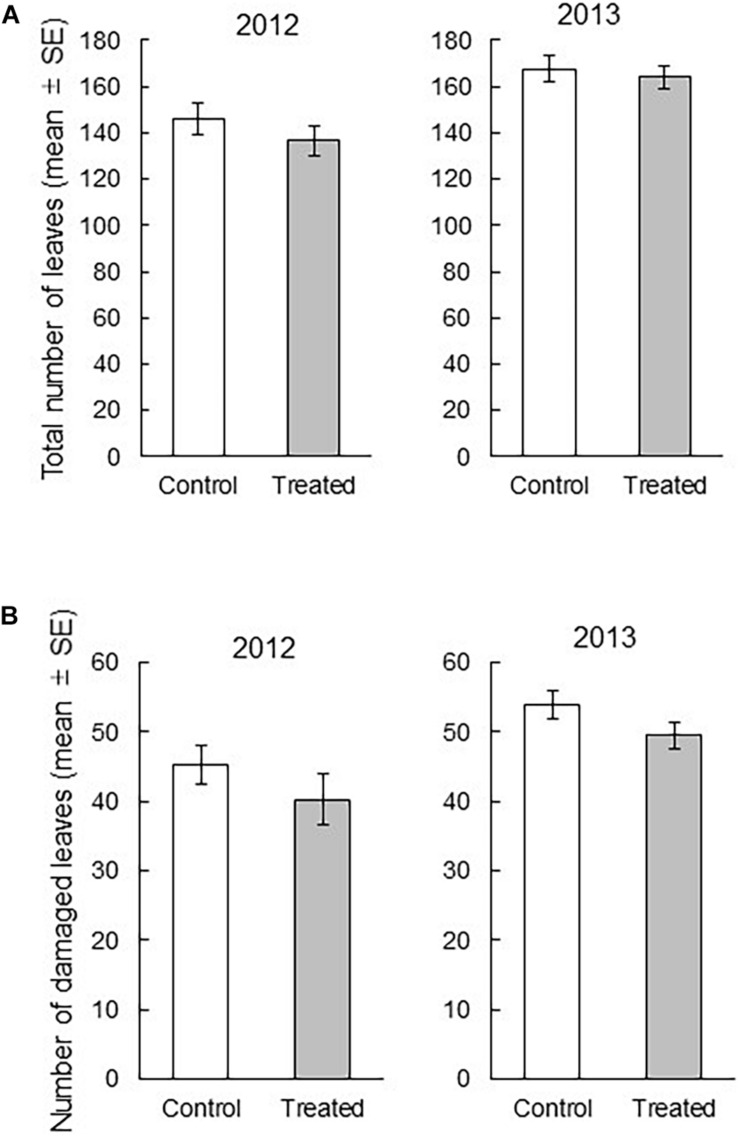
**(A)** Comparison between the total number of leaves in a treated bunch and that in a control bunch (treatment *P* = 0.3147, year *P* = 0.0001, treatment × year *P* = 0.7884), and **(B)** the comparison between the total number of damaged leaves in a treated bunch and that in a control bunch (treatment *P* = 0.0468, year *P* = 0.0003, treatment × year *P* = 0.8564). The effects of treatment, year, and their interaction on the number of leaves were analyzed with the least square mixed model after Box-Cox transformation. *N* = 15, except for the data of one field in 2013 (*N* = 14).

We found that the treatment and the interaction of treatment × year did not significantly influence the total number of leaves per bunch (treatment: df = 1, 159.6, *F* = 1.0171, *P* = 0.3147; interaction: df = 1, 7.138, *F* = 0.0777, *P* = 0.7884), whereas the year had highly significant influence on the total number of leaves per bunch (df = 1, 159.6, *F* = 15.2639, *P* = 0.0001) (Figure 1A). In contrast, we found that the treatment and year had significant influence on the number of damaged leaves per bunch (treatment: df = 1, 173.2, *F* = 4.008, *P* = 0.0468, year: df = 1, 173.2, *F* = 13.3238, *P* = 0.0003), whereas their interaction did not (df = 1, 6.763, *F* = 0.0354, *P* = 0.8564). These results indicated that over 2 years, the number of damaged leaves was significantly lower in the exposed rice plants than in the control rice plants (Figure 1). The relative decrease of the damaged leaves in the exposed plants compared to that in control plants of respective year was 11.1% in 2012 and 9.8% in 2013.

### Grain Weight

**FIGURE 2:**
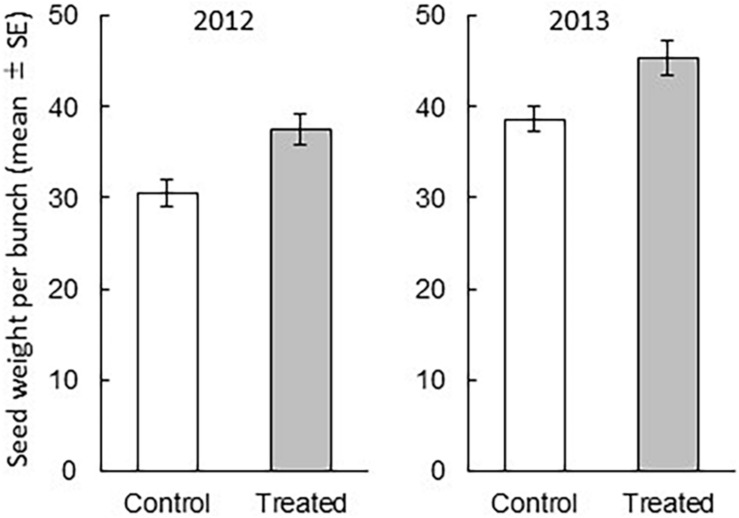
Comparison between the rice grain weight (mean ± SE) from a treated bunch and that from a control bunch (treatment *P* = 0.0073, year *P* < 0.0001, treatment × year *P* = 0.9126). The effects of treatment, year, and their interaction on grain weight per bunch were analyzed with the least square mixed model after Box-Cox transformation. *N* = 15, except for the data of one field in 2013 (*N* = 14).

We found that the treatment and year had significant influence on the grain weight per bunch (treatment: df = 1, 174, *F* = 7.3799, *P* = 0.0073, year: df = 1, 174, *F* = 16.2277, *P* < 0.0001), whereas their interaction did not (df = 1, 5.838, *F* = 0.0131, *P* = 0.9126) (Figure 2). In addition, we measured the weight of 100 grains to obtain the weight of one rice grain. In both years, the weight of a grain from treated bunches (mean ± SE) was not significantly different from that from the control bunches (2012: the control bunch = 2.683 ± 0.026; the treated bunch = 2.659 ± 0.028, *P* = 0.476; 2013: the control bunch = 2.684 ± 0.024; the treated bunch = 2.633 ± 0.033, *P* = 0.251). These results indicated that over 2 years, the weight of rice grains per bunch was significantly higher in the treated rice plants than in the control rice plants. The increase in grain weight per bunch was explained by the increase of the number of grains. The relative increase of the number of grains in the exposed plants were 23% in 2012 and 15.9% in 2013 compared to that in control rice plants of respective year.

### Chemical Analyses

The headspace of volatiles emitted from the five odor sources was analyzed separately. We found 26 compounds in all the odor sources (Table 1). The compounds were classified into monoterpenoids (12 compounds, 40.4% in total), green leaf volatiles (eight compounds, 46.5%), short-chain (C5) alcohols (two compounds, 5.3%), short-chain (C5) ketone (5.4%), short-chain (C5) acetate (0.5%), short-chain (C9) aldehyde (1.1%), and hydrocarbon (0.7%). The major compounds were (*Z*)-3-hexen-1-ol, (*Z*)-3-hexenyl acetate, α-pinene, camphene, β-pinene, β-myrcene, 1,8-cyneole, and pentene-3-one.

**TABLE 1:** The volatile organic compounds in the headspace of artificially damaged weed.

Compounds	Peak area/gFW (× 10^–3^)
**Alcohols**	
1-penten-3-ol	3,086 ± 244
(*Z*)-2-penten-l-ol	492 ± 60
(*Z*)-3-hexen-l-ol	14,963 ± 1,381
(*E*)-2-hexen-l-ol	2,146 ± 598
hexan-1-ol	1,004 ± 109
**Aldehydes**	
(*Z*)-3-hexanal	3,760 ± 800
Hexanal	713 ± 44
(*E*)-2-hexenal	1486 ± 414
nonanal	731 ± 126
**Acetates**	
(*Z*)-2-penten-1-yl acetate	364 ± 39
(*Z*)-3-hexen-1-yl acetate	6,692 ± 634
(*E*)-2-hexen-l-yl acetate	722 ± 95
**Terpenoids**	
tricyclene	538 ± 155
α-thujene	373 ± 52
α-pinene	6,336 ± 1,222
camphene	5,408 ± 1,368
β-pinene	5,975 ± 1,570
β-myrcene	2,280 ± 557
α-phellandrene	534 ± 250
α-terpinene	826 ± 175
limonene	1,478 ± 284
1,8-cineole	2,300 ± 830
γ-terpinene	955 ± 463
terpinolene	357 ± 69
**Others**	
pentan-3-one	3,670 ± 429
1-octene	494 ± 216

*Data are represented by mean ± standard error.*

## Discussion

Artificially damaged weeds set in the seedling bed emitted 26 volatile compounds, including green leaf volatiles (GLVs), monoterpenoids, short-chain (C5) alcohol/ketone/acetate, a short-chain (C9) aldehyde, and a hydrocarbon (Table 1). The exposure of rice seedlings to these volatiles resulted in a significant increase in the number of rice grains (23% increase in 2012 and 18% increase in 2013 compared to that in control rice plants). It is important to note that the exposure was only three times with an interval of 4 days at the seedling stage (from ca. 5 cm to ca. 10 cm plant height), indicating that the exposure at the rice seedling stage affected the rice grain production.

One of the possible reasons for the increase would be the reduction of the leaf damages in the exposed plants. Although the damaged leaves of the exposed rice plants were significantly lower than that of the control rice plants, the differences were relatively small (11.1% reduction in 2012 and 9.8% reduction in 2013). In this study, we did not evaluate the behavior of the herbivorous insects observed in the rice paddy fields. The mechanisms involved in the reduction of the damage made by herbivores insects remains necessary.

The differences in the rice grain production between the control and treated plants might also be explained by other factors, such as effects of fungi/viruses, growth, nutrition intake, photosynthesis, which might be affected by the volatiles either positively or negatively. As we did not find disease symptoms on leaves, and the farmer did not mention to us disease problems during the experimental periods, the involvement of fungi and viruses was unlikely. Whether other factors were involved in the increase of performance in rice plants is worth evaluating, as previous studies showed that volatiles affect plant physiology. (*E*)-2-Hexenal acts as signal chemicals that strongly induce the gene expression of abiotic-related transcription factors in *Arabidopsis* (Yamauchi et al., 2015). Inhibitory effects on the growth of volatile compounds have been reported in *Arabidopsis*, for example, on root length in *Arabidopsis* (Bate and Rothstein, 1998; Mirabella et al., 2008; Scala et al., 2017). Horiuchi et al. (2007) also reported that monoterpenoids, borneol, and bornyl acetate reduced the root length of *Arabidopsis*.

The origins and the principal compounds involved in the increased rice grain production remained unknown. Among them, GLVs are commonly found in green plants whose leaves underwent mechanical damage. GLVs have been reported as signaling molecules in the elicitation of priming in several plant species (Bate and Rothstein, 1998; Gomi et al., 2003; Engelberth et al., 2004; Farag et al., 2005; Kishimoto et al., 2005, 2006; Paschold et al., 2006; Yamauchi et al., 2018). Volatile terpenoids are also involved in the priming. Soybean plants previously exposed to a blend of α-pinene, β-myrcene, and limonene became more defensive against common cutworms (*Spodoptera litura* larvae) (Shiojiri et al., 2017). 1.8-Cineol, constitutively released from mint plants (*Mentha* × *piperita*), was suggested to be one of the signaling molecules that induce resistant responses in soybean plants (Sukegawa et al., 2018). Riedlmeier et al. (2017) reported that a mixture of α-pinene and β-pinene induced resistant responses in *Arabidopsis*.

Detailed studies on the relationship between the effects of the volatile exposure to rice seedlings and the increased production of rice grains, especially focusing on the mechanism involved in the long-lasting effects, are needed. The effects of the timing of the exposure (i.e., which growth stages are most effective) should also be considered. Further, for achieving an effective exposure-related increase of rice grain production, it is worthwhile to evaluate whether specific weed groups (specific families or genera) are needed for the exposure, or if any weeds in the areas surrounding rice paddy fields can be used for the exposure. Thus, our findings suggest that exposure of volatiles from mechanically damaged weeds to rice seedlings has the potential to increase rice production.

## Data Availability Statement

The original contributions presented in the study are included in the article/Supplementary Material, further inquiries can be directed to the corresponding author/s.

## Author Contributions

KS designed and conducted the field experiments. RO conducted the field experiments and the chemical analyses. MU conducted the statistical analyses. KS, RO, MU, and JT analyzed the data and wrote the manuscript. All authors gave final approval of the manuscript for publication.

## Conflict of Interest

The authors declare that the research was conducted in the absence of any commercial or financial relationships that could be construed as a potential conflict of interest.
