# Supplementary material for: Field-Grown Rice Plants Become More Productive When Exposed to Artificially Damaged Weed Volatiles at the Seedling Stage
Source: Front Plant Sci. 2021 Jul 12;12:692924. doi: 10.3389/fpls.2021.692924 (PMC8312646; doi:10.3389/fpls.2021.692924)
Supplement: Supplementary file 1 [file Table_1.pdf]

Supplemental Table 1. The experimental protocol in 2012 and 2013

	Year	
	2012	2013
Treatment days	May 3rd , 7th, 11th	May 12th,16th, 20th
Transplantation days to the paddy fields	May 14th and May 21st	May 23rd and May 26th
Damage investigation	July 19th	July 18th
Harvesting	September 12th	September 18th
